# Clinical features of rabies patients with abnormal sexual behaviors as the presenting manifestations: a case report and literature review

**DOI:** 10.1186/s12879-019-4252-4

**Published:** 2019-08-01

**Authors:** Zhaoxing Tian, Yingyu Chen, Wei Yan

**Affiliations:** 1 Department of Emergency Medicine, Peking University International Hospital, 1 Life Park Road, Zhongguancun Life Science Park, Changping District, Beijing, 102206 China; 2 Division of Education, Beijing Jishuitan Hospital, 31 Xinjiekou East Street, Xicheng District, Beijing, 100035 China; 3 Department of Respiratory Medicine, Peking University Third Hospital, 49 North Garden Road, Haidian District, Beijing, 100191 People’s Republic of China

**Keywords:** Rabies, Sexual behavior, Ejaculation, Hypersexuality, Early diagnosis, Survival

## Abstract

**Background:**

Abnormal sexual behaviors presenting as manifestations of rabies have occasionally been reported in the literature, although little attention has been paid to these cases to date. This study aimed to analyze the clinical features of rabies cases with abnormal sexual behaviors as the presenting manifestations.

**Case presentation:**

A case of 32-year-old man with frequent ejaculation as the initial symptom of rabies was first reported. Then, a literature review was conducted using databases including CNKI, SinoMed, VIP, Wanfang Data, ScienceDirect, ProQuest, OVID and PubMed. In addition to our case, 54 other rabies cases, with abnormal sexual behaviors as the presenting manifestations, have been reported since 1970. Among the 55 cases, 51 were male and three were female (unknown gender for one case), with ages ranging from 6 to 71 years. All cases were reported in developing countries, 46 in China. Dog bites were the major source of infection, and extremities were the main exposure sites. Overall, 46 (83.6%) cases had abnormal sexual behaviors as the initial symptoms. The major presenting manifestations were priapism and ejaculation in males and hypersexuality in females. All cases were clinically diagnosed based on medical history and clinical manifestations. Given no standardized post-exposure prophylaxis, all cases died with the survival time being between 1 and 15 days.

**Conclusions:**

The rabies patients with abnormal sexual behaviors have unique clinical features. To avoid misdiagnosis, unexplained abnormal sexual behaviors should raise clinical suspicion of rabies.

## Background

Rabies is an acute zoonotic infectious disease caused by the rabies virus that severely impacts the central nervous system. Rabies is commonly seen in carnivorous animals such as dogs, wolves, cats, and bats, and the virus is transmitted by a bite from an infected animal [1, 2]. The mortality of rabies is extremely high; an unvaccinated infected person is expected to live for only a maximum of 7 days after the appearance of symptoms if timely and appropriate therapy is not applied. It is estimated that there are at least 55,000 deaths per year worldwide from rabies; about 56% of these deaths occur in Asia and 44% in Africa (particularly in rural areas of both continents), and almost all of these patients missed the narrow window for optimal treatment due to misdiagnosis [1–3].

Typical symptoms of rabies include aggression; hydrophobia; anemophobia; progressive paralysis; and hypersensitivity to sound, light, wind, and pain. In the early stage, atypical flu-like symptoms such as fever, loss of appetite, nausea, headache, fatigue, and general malaise may also appear. Moreover, uncommon symptoms and signs reflecting abnormal sexual behaviors, including frequent ejaculation, priapism, hypersexuality, and other abnormal sexual behaviors, may be the representing manifestations of rabies, which may lead to misdiagnosis. Indeed, over the last five decades, more than 50 sporadic cases with atypical early manifestations have been reported worldwide [4–40]; most cases were misdiagnosed or not diagnosed at all, and all resulted in death.

In 2014, we admitted a male patient presenting with frequent ejaculation, and a diagnosis of rabies was confirmed. Unfortunately, many physicians are not aware of these atypical cases, and do not consider rabies when encountering a patient presenting with a change in sexual behavior in clinical practice. Considering the high mortality and strong infectivity, this paper will systematically analyze and summarize the clinical features of abnormal sexual behaviors as the presenting manifestations in patients with rabies, by first reporting our case and then presenting data derived from a literature search.

## Case presentation

A 32-year-old Chinese man began to have frequent ejaculations on December 7 (day 1), 2014, at a frequency of 5–6 times a day; these reached 20–30 times on day 3. When frequent ejaculation increased to 40–50 times on the morning of day 4, the patient went to a local clinic in Beijing that specialized in traditional Chinese medicine for treatment of “imbalance of *Yin* and *Yang*”. However, symptomatic treatment to rebalance *Yin* and *Yang* had no effect. In the same afternoon, he was sent to a community hospital in Beijing with the following symptoms: headache, dizziness, nausea, and malaise; fever of 39 °C; irritability; tachyphrasia; speech difficulty; and hypersalivation. He was subsequently transferred to a tertiary hospital in Beijing for further diagnosis and treatment, but the etiology remained unidentified. At around 10:00 pm on day 4, the patient was sent to the Infectious Disease Department of Peking University Third Hospital, and then transferred to the Emergency Department due to tachycardia and dyspnea. His complaints included high penis sensitivity, painful erections, and ejaculations > 40 times a day triggered by any touch (or ejaculations without erection and release of semen) as well as headache, nausea, chest congestion, and fever. There was no significant improvement after fluid infusion, symptomatic treatment, and other supportive therapies. No diagnosis was made even after consulting urologists, neurologists, and psychiatrists until 5:00 pm on day 5 when rabies was finally considered as typical symptoms such as anemophobia, hydrophobia, and photophobia then emerged. After questioning the patient and his family, he admitted a history of a scratch on the right foot caused by a dog about 4 months earlier. The wound was superficial and left untreated, and neither rabies vaccine nor a passive immune preparation was given. At 10:30 am on day 6, he developed apnea and was intubated, and multiple vasopressors were given at the same time. His condition continued to deteriorate rapidly, and he was declared clinically dead at 12:59 pm the same day.

Two days after the death of the patient, positivity of rabies virus neutralizing antibody (the exact titer was not available due to the absence of quantitative analysis) in serum samples, and a positive lyssavirus signal in a polymerase chain reaction targeting the conserved region of the nucleoprotein gene of lyssaviruses in two saliva specimens were reported by Beijing Center for Disease Control and Prevention (http://www.bjcdc.org/article/39579/2014/12/2014122339579.html) Thus, the diagnosis of rabies was officially established. The patient’s family refused the recommendation for autopsy.

## Literature review

A literature search of papers reporting rabies cases with abnormal sexual behaviors (or abnormal sexual symptoms as the presenting manifestations) was performed on December 31, 2017. Chinese keywords, (“rabies” or “hydrophobia”) and (“abnormal sexual behaviors”, “frequent ejaculation”, “priapism”, or “hypersexuality”) from databases including CNKI, SinoMed, VIP and Wanfang Data, and English keywords, (“rabies”, “canine madness”, “Lyssa”, “rabid”, “lupomania”, “hydrophobia”, “photophobia”, “photopsia”, or “aerophobia”) and (“sexual arousal”, “sexual desire”, “hypersexual behavior”, “orgasm”, “hypersexuality”, “urogenital symptom”, “libido”, “sexual manifestation”, “priapism”, “hyperlibidinism”, “penile hyperexcitability”, “erection”, or “ejaculation”) from databases, including ScienceDirect, ProQuest, OVID and PubMed, were searched. Relevant references of the searched papers were also checked carefully. Then the literature on rabies cases including patient demographic data, medical history, symptoms, signs, diagnosis, treatment, and outcome were identified and analyzed.

A total of 47 papers published between 1970 and 2015, including 54 rabies cases, were identified that reported abnormal sexual behaviors as the presenting manifestations [4–50]. These cases, along with the case we present here, were then reviewed. Among these 47 papers, 38 were published in Chinese reporting 45 cases, and nine papers were published in English reporting nine cases (Table 1).

**Table 1:** Reported cases of rabies with abnormal sexual behaviors as the presenting manifestations

Authors (Reference)	Published year	Country	Case (sex, age)	Sexual manifestation	Duration of the manifestation	Diagnosis methods
Gardner [42]	1970	Burma	1 (M, NR)	Desperate increase in libido	NR	Negri bodies were indeed seen on section of this patient’s hippocampus
Talaulicar [44]	1977	India	1 (M, 25Y)	Persistent priapism^#^	1.5D	Medical history, clinical manifestation, dog was proved rabid.
Bhandari & Kumar [7]	1986	India	1 (M, 56Y)	Frequent erection and ejaculation > 10 times a day, penile hyperexcitability^#^	2D	Medical history and clinical manifestation
Jiang, et al [11]	1986	China	1 (M, 41Y)	Urgent urination, urinary endless and spermatorrhea^#^	4D	Medical history and clinical manifestation
Wang, et al [23]	1987	China	1 (M, 40Y)	Priapism, spermiation^#^	15D	Medical history and clinical manifestation
Madhusudana et al [43]	1988	India	1 (M, 34Y)	Increased libido, frequent erection and ejaculation > 10 times a day^#^	NR	Medical history and clinical manifestation, corneal smears were positive for rabies antigen by fluorescent antibody technique
Udwadia et al [8]	1988	India	1 (M, 47Y)	Frequent erection and ejaculation > 10 times a day^#^	2D	Medical history, clinical manifestation, Negri bodies in brain tissue by autopsy, and positive result of Swiss albino mouse inoculation test
Wang, et al [38]	1989	China	2 (2 M, 32Y & 44Y)	Frequent erection and ejaculation > 10 times a day^#^	5D & 6D	Medical history and clinical manifestation
Lin, et al [20]	1989	China	1 (M, 52Y)	Spermatorrhea > 10 times, painful penis^#^	3D	Medical history and clinical manifestation
Liu, et al [12]	1990	China	1 (M, 65Y)	Frequent erection and ejaculation > 50 times a day^#^	4D	Medical history and clinical manifestation
Xiao, et al [15]	1990	China	1 (M. 41Y)	Frequent erection and ejaculation > 50 times a day^#^	4D	Medical history and clinical manifestation
Luo, et al [35]	1990	China	1 (M, 35Y)	Frequent erection and ejaculation > 10 times a day^#^	4D	Medical history and clinical manifestation
Zhang, et al [37]	1990	China	1 (M, 35Y)	Frequent erection and ejaculation > 20 times a day^#^	5D	Medical history and clinical manifestation
Yang, et al [25]	1990	China	1 (M, 33Y)	Frequent spermatorrhea	3D	Medical history and clinical manifestation
Ma, et al [17]	1991	China	1 (M, 35Y)	Frequent erection and ejaculation > 20 times a day	2D	Medical history and clinical manifestation
Fu, et al [19]	1991	China	1 (M, 28Y)	Priapism > 50 times a day	3D	Medical history and clinical manifestation
Zhan, et al [32]	1991	China	1 (M, 30Y)	Priapism and spermatorrhea 5–10 times^#^	4D	Medical history and clinical manifestation
Chou et al [31]	1991	China	1 (M, 46Y)	Frequent spermatorrhea^#^	11D	Medical history and clinical manifestation
Ge, et al [16]	1992	China	2 (2 M, 40Y & 56Y)	Frequent erection and ejaculation > 10 times a day^#^	4D & 6D	Medical history and clinical manifestation
Xiao, et al [27]	1992	China	1 (M, 62Y)	Frequent erection and ejaculation > 10 times a day^#^	8D	Medical history and clinical manifestation
Wei, et al [30]	1992	China	1 (M, 61Y)	Frequent erection and ejaculation > 50 times a day^#^	4D	Medical history and clinical manifestation
Niu, et al [14]	1993	China	1 (M, 68Y)	Frequent erection and ejaculation > 20 times a day^#^	4D	Medical history and clinical manifestation
Feng, et al [21]	1994	China	1 (F, 45Y)	Feeling of ants climb in vagina^#^	2D	Medical history and clinical manifestation
Geng, et al [36]	1994	China	1 (M, 59Y)	Frequent erection and ejaculation > 10 times a day^#^	7D	Medical history and clinical manifestation
Li, et al [45]	1994	China	1 (M, 52Y)	Frequent erection and ejaculation < 10 times a day^#^	7D	Medical history and clinical manifestation
He, et al [34]	1994	China	1 (M, 65Y)	Pain of penis^#^	6D	Medical history and clinical manifestation
Li, et al [26]	1994	China	1 (M, 32Y)	Priapism and ejaculation > 10 times^#^	3D	Medical history and clinical manifestation
Tang, et al [24]	1994	China	1 (M, 27Y)	Frequent erection and ejaculation > 10 times a day	5D	Medical history and clinical manifestation
Zhang, et al [13]	1995	China	1 (M, 34Y)	Frequent erection and ejaculation > 10 times a day^#^	5D	Medical history and clinical manifestation
Dutta [5]	1996	India	1 (F, 28Y)	Hypersexuality^#^	10D	Medical history, clinical manifestation and Negri bodies in brain tissue by autopsy
Cai, et al [29]	1997	China	2 (2 M, 29Y & 31Y)	Frequent erection and ejaculation > 50 times a day^#^	8D & 6D	Medical history and clinical manifestation
Yang, et al [18]	1998	China	1 (M, 40 + Y)	Priapism	1D	Medical history and clinical manifestation
Du, et al [22]	1999	China	1 (M, 26Y)	Frequent erection and ejaculation > 20 times a day^#^	10D	Medical history and clinical manifestation
Wu, et al [28]	2000	China	5 (5 M, 18-52Y)	Frequent erection and ejaculation > 10 times a day^#^	5-7D	Medical history and clinical manifestation
Zhong, et al [33]	2000	China	1 (M, 54Y)	Frequent erection and Ejaculation > 11 times a day^#^	7D	Medical history and clinical manifestation
Li, et al [40]	2000	China	1 (M, 39Y)	Frequent erection and ejaculation > 30 times a day^#^	4D	Medical history and clinical manifestation
Ou, et al [46]	2005	China	1 (M, 35Y)	Frequent erection and ejaculation > 10 times a day^#^	6D	Medical history and clinical manifestation
Daher, et al [41]	2005	Brazil	1 (NR, NR)	Priapism	NR	Clinical manifestations, laboratory tests and postmortem findings
Tan, et al [10]	2007	China	1 (M, 71Y)	Severe pain of scrotum and testicles^#^	6D	Medical history and clinical manifestation
Li, et al [39]	2011	China	1 (M, 43Y)	Priapism and ejaculation < 10 times a day^#^	2D	Medical history and clinical manifestation
Liu, et al [47]	2011	China	1 (M, 8Y)	Priapism and ejaculation 0 times^#^	6D	Medical history and clinical manifestation
Senthilkumaran et al [6]	2011	India	1 (F, 28Y)	Hypersexuality, frequent intercourse and frequent orgasm^#^	6D	Medical history, clinical manifestation, imaging, CSF PCR and skin biopsy
Depani & Molyneux [4]	2012	Malawi	1 (M, 6Y)	Priapism with pain^#^	1D	Medical history and clinical manifestation
Qin, et al [48]	2012	China	1 (M, 52Y)	Priapism and ejaculation < 10 times a day^#^	3D	Medical history and clinical manifestation
Lei, et al [9]	2012	China	1 (M, 53Y)	Priapism	2D	Medical history and clinical manifestation
Liu, et al [49]	2013	China	1 (M, 48Y)	Priapism	2D	Medical history and clinical manifestation
Gao, et al [50]	2014	China	1 (M, 66Y)	Priapism and ejaculation > 5 times a day^#^	8D	Medical history and clinical manifestation
Yan, et al.	Submitted	China	1 (M, 32Y)	Frequent erection and ejaculation > 40 times a day^#^	6D	Medical history and clinical manifestation

*NR* Not reported, *M* Male, *F* Female, *Y* Years, *D* Days

^#^ Abnormal sexual behaviors as the initial manifestations

## Analysis of cases

Among the 55 cases, including our case, 51 were male and three were female, with ages ranging from 6 to 71 years for males and 28 to 45 years for females; the gender of one case was not reported. All cases were reported in developing countries; 46 (83.6%) in China, six (10.9%) in India, one (1.8%) in Burma, one (1.8%) in Brazil and one (1.8%) in Malawi. Animal bites were the major source of infection with 51 (92.7%) cases being infected by dogs, one by a cat, one by a mouse, one by a mongoose, and one by an unspecified source. Extremities were the main exposure (bite) sites, accounting for 43 (78.2%) cases: 30 cases suffered bites to the lower extremities (i.e. thighs, legs, ankles, and feet), 12 in the upper extremities (i.e. arms and hands), and one in both upper and lower extremities. Other exposure sites included the face (*n* = 1) and unspecified sites (*n* = 11) (Table 2).

**Table 2:** Summary of clinical features of 55 cases of rabies with abnormal sexual behaviors as the presenting manifestations

Variables	N (%)
Sex	
Male	51 (92.7%)
Female	3 (5.5%)
Unknown	1 (1.8%)
Age (years) ^a^	
Range	6–71
Median	40
Source of infection	
Dog	51 (92.7%)
Cat	1 (1.8%)
Mouse	1 (1.8%)
Mongoose	1 (1.8%)
Unknown	1 (1.8%)
Exposure site	
Leg	25 (45.5%)
Arm	10 (18.2%)
Leg and arm	1 (1.8%)
Feet or ankles	5 (9.1%)
Hand	2 (3.6%)
Close contact	1 (1.8%)
Face	1 (1.8%)
Unknown	10 (18.2%)
Incubation period (months) ^b^	
Range	1–24
Median	3
Diagnostic methods	
Animal bite	5 (9.1%)
Typical symptoms	1 (1.8%)
Animal bite + typical symptoms	42 (76.4%)
Animal bite + typical symptoms + virological or other techniques	7 (12.7%)
Typical symptoms of rabies	
Yes	50 (90.9%)
No	5 (9.1%)
Abnormal sexual behaviors^d^	
*Male (n = 51)*	
Frequent ejaculation	35 (68.6%)
< 10	4 (11.4%)
10–19	20 (57.1%)
20–49	6 (17.1%)
≥ 50	5 (14.3%)
Spermatorrhea	6 (11.8%)
Pain of penis	3 (5.9%)
Pain of scrotum and testicles	1 (2.0%)
Priapism or frequent erection	45 (88.2%)
Hypersexuality or increased libido	2 (3.9%)
*Female (n = 3)*	
Hypersexuality, frequent intercourse and frequent orgasm	1 (33.3%)
Hypersexuality	1 (33.3%)
Feeling of ants climbing in vagina	1 (33.3%)
*Gender unknown (n = 1)*	
Priapism	1
Abnormal sexual behaviors as initial symptoms	
Yes	46 (83.6%)
No	8 (14.5%)
Unknown	1 (1.8%)
Duration of abnormal sexual behaviors (days) ^c^	
Range	1–15
Median	4
Debridement and vaccination after exposure	
Debridement	1 (1.8%)
Vaccination	2 (3.6%)
Debridement with vaccination	1 (1.8%)
None	49 (89.1%)
Unknown	2 (3.6%)
Outcome	
Death	55 (100%)
Survival	0 (0%)

Data are expressed as the number (%), unless otherwise indicated

^a^, information on the exact ages was available for 48 cases; ^b^, information on the exact incubation period was available for 49 cases; ^c^, information on the exact duration of abnormal sexual behaviors was available for 47 cases; ^d^, One patient may have more than one abnormal sexual behaviors

The associated abnormal sexual behaviors are shown in Table 2. The major presenting manifestations were priapism, ejaculation, and spermatorrhea in male patients and nymphomania, hypersexuality, and other abnormal sexual behavior in female patients. Overall, 46 (83.6%) of the 55 cases had abnormal sexual behaviors as the initial presenting symptoms. The duration of abnormal sexual behaviors ranged from 1 day (2 cases) to 15 days (1 case), with a median of 4 days and a mean of 5.0 days.

No patients received any standardized post-exposure prophylaxis. Among the 53 cases with available information on vaccination after exposure, only one case received debridement and 5-dose rabies vaccine immediately after biting [10]. Moreover, only two cases received rabies vaccination: one received one dose [15] while the other case received three doses [33], and one unvaccinated case was given debridement immediately after being bitten [8].

The rabies incubation period ranged from 1 month to 24 months and was mostly between 2 months and 24 months. Among the 54 cases for whom detailed descriptions on the presenting manifestations were available, abnormal sexual behaviors were the initial manifestations in 46 (85.2%), and were the concurrent manifestations in eight (14.8%) cases. All, except for five, cases [28] developed typical symptoms of rabies, including hydrophobia, anemophobia, and photophobia.

Rabies was clinically diagnosed in all, but five [28], of the cases based on medical history and clinical manifestations; the diagnosis was confirmed by virological or other techniques, such as brain pathology or imaging only for seven cases [5, 6, 8, 40–43]. In the report by Wu et al., all five patients were bitten by dogs and did not receive debridement and vaccination, and priapism and frequent ejaculation were the initial presenting symptoms, without the development of typical manifestations of rabies or abnormal laboratory findings.

A rabies diagnosis was immediately made for all cases with typical rabies symptoms and abnormal sexual behaviors being the concurrent presenting manifestations. However, the diagnosis was not made for all cases with abnormal sexual behaviors alone as the initial presenting manifestations (before typical rabies symptoms appeared) and thus these cases were misdiagnosed prior to the appearance of typical symptoms.

All of the rabies patients died after the appearance of the presenting symptoms, with the survival time (duration between appearance of the presenting symptoms and death) ranging from 1 to 15 days.

## Discussion and conclusions

Rabies is an uncommon but deadly disease, with aggression, hydrophobia, anemophobia, and progressive paralysis being its typical symptoms [1, 2]. However, in addition to flu-like symptoms, abnormal sexual behaviors such as priapism, frequent ejaculation, and hypersexuality also occasionally appear as initial or concurrent presenting atypical symptoms. Since the first published report from Burma of a male rabies case with greatly increased libido, 54 cases with abnormal sexual behaviors have been reported in the literature worldwide (mainly from China and India).

In the present review, we added one more case with frequent ejaculation as the initial presenting symptom of rabies. All of these cases, with abnormal sexual behaviors as the initial symptoms, were misdiagnosed in the early phase. Among these cases, 46 (85.2%) patients had abnormal sexual behaviors as the initial symptoms. The correct diagnosis was made only in the later stage of all cases when typical rabies symptoms appeared through medical history and clinical manifestations; however, the diagnosis was confirmed by virological, postmortem pathological testing and/or imaging examination only in a few cases.

It is noticeable that among the 53 cases with abnormal sexual behaviors of rabies and reported ages, 51 (96.2%) were 18 or older and two (3.8%) under 10 (they were 6 and 8 years old respectively). Also, among the 54 cases whose gender was reported, 51 (94.4%) were males and only three (5.6%) were females. These findings suggest that abnormal sexual behaviors of rabies mostly occur in adult males; however, the symptoms can occasionally occurs in boys under 10 and in adult females.

After examining the cases in the literature and in this review, we identified the following reasons as likely for the misdiagnoses: (1) rabies with abnormal sexual behaviors as the initial symptoms is clinically rare, and many physicians in general medicine, emergency medicine, urology and even psychiatry have insufficient knowledge or lack of awareness about abnormal sexual manifestations of rabies; (2) inability to remember details or avoidance by patients and their family to provide medical history may influence the initial establishment of diagnosis; (3) physicians fail to strictly follow treatment guidelines; they neither carefully elicit a medical history and course of disease nor seriously analyze the correlation between symptoms; and (4) rabies is not considered, simply because some physicians falsely believe that rabies is extremely rare clinically, or has already been eradicated in developing countries (it has only been eradicated in some developed countries) [2].

Once exposure occurs, timely post-exposure prophylaxis, including correct wound care and appropriate vaccination, is believed to effectively prevent the progression to clinical disease [51]. However, rabies continues to cause about 61,000 human deaths every year globally [52], indicating that this disease remains to be underestimated and the true burden of this disease has not been captured [51]. The primary reason for these miserable deaths is that a large number of victims do not receive rabies vaccination at all, and some of those who do do not complete the full course. Noticeably, while rabies used to be considered certainly fatal, several rabies survivors have been increasingly reported over the past two decades worldwide, particularly in recent years [53–61]. Excellent intensive care facilities and aggressive management approaches with appropriate supportive care are believed to contribute to the increase of human rabies survivors [53]. For example, in India, although public health facilities may be lacking (especially in rural areas), several private and some public medical institutes provide world-class medical care, thus likely playing an important role in the prolonged survival of human rabies patients [53]. Most recently, Mani et al. [61] reported two cases whose lives were saved after receiving both rabies vaccine and immunoglobulin and intensive care. However, the patients progressed to clinical disease with typical symptoms of rabies, probably due to multiple exposures or bites on highly innervated areas [61].

Therefore, we believe that if the treating physicians try to confirm the intra vitam diagnosis, and antemortem laboratory facilities such as virological test, imaging examination, or other techniques are readily available, then the rabies diagnosis can be made in the early stage, and timely aggressive treatment and care would significantly increase the chance for the patient to survive. Indeed, in 2005, Willoughby et al. reported that a 15-year-old girl almost completely recovered from rabies after being in an induced coma while the native immune response matured but rabies vaccine was not administered [62]; this has provided an impetus for physicians to attempt aggressive management and given hope for rabies patients to survive. Therefore, better awareness among physicians that rabies patients may present with abnormal sexual symptoms as initial presenting manifestations and the fact that rabies patients with atypical manifestations may recover prior to the appearance of typical symptoms is clinically important for those working in general medicine, emergency medicine, urology, and psychiatry.

Nevertheless, it must be emphasized that most of the reported human rabies survivors did not achieve a complete recovery and they developed various neurological sequelae. Mani et al. reported eight patients with laboratory-confirmed rabies who were managed with supportive care in intensive care units and finally survived [61]. Unfortunately, all except for one case had moderate to severe neurological sequelae with poor functional outcomes [61]. Moreover, many efforts have been made to replicate the expensive and intensive “Milwaukee protocol” [62], but generally been unsuccessful. In a tertiary care hospital in South India, Manesh et al. reported that the attempts to treat three patients of canine-acquired rabies encephalitis using similar protocol all turned out to be failed [63]. Hence it is urgent to find novel antiviral drugs and innovative therapeutic strategies to improve the outcomes, particularly in relation to neurological sequelae.

The pathogenesis of abnormal sexual behaviors in patients with rabies has not been fully elucidated. The following mechanisms have been proposed. After exposure, the strongly neurotropic rabies virus stays and replicates in the invasion site as well as in the nerve fibers of muscle spindle receptor in nearby striated muscle cells. Then the virus travels centrally along peripheral nerves and massively proliferates at dorsal root ganglia. When the rabies virus invades lumbosacral cord segments, nerve irritation impulses lead to penile erection in males. Nerve impulses travel in the smooth muscles of ductus deferens, seminal vesicle, prostate, and ischiocavernosus muscle and through the hypogastric nerve and sympathetic nerve of the hypogastric plexus thus causing rhythmic contraction of this muscle group, which may result in ejaculation [8, 28]. In females, nymphomania or hypersexuality may be present due to the influence of nerve impulses from involved lumbosacral cord segments on sexual organs [5, 6]. In addition to the effect on the spinal cord that may produce sexual excitement, the rabies virus can also interfere with the function of the hypothalamus, amygdaloid nucleus, and limbic system, resulting in hypersexuality which may present earlier than other typical symptoms such as hydrophobia and anemophobia [5, 6, 8].

Physicians should be mindful that many diseases can present with abnormal sexual behaviors, especially priapism which can be seen in leukemia, infection, central nervous system diseases, spinal injury, penis injury, or tumor metastasis, and may be related to antipsychotic drugs, sickle cell anemia, and penile dorsal vein embolization [64]. However, the absence of ejaculation and psychiatric symptoms in most non-rabies cases is a useful feature for the differential diagnosis. Finally, we should remember that in addition to flu-like symptoms and abnormal sexual behaviors, patients with rabies may also present other atypical clinical symptoms, such as acute disseminated encephalomyelitis, impaired vagus ganglion, sympathetic ganglia, and cardiac ganglion, which can lead to dysfunction of the heart and cardiovascular system, and even sudden death [2, 65].

In conclusion, patients with abnormal sexual behaviors as presenting manifestations have unique clinical features. It is important to improve the level of awareness of rabies with abnormal sexual behaviors as presenting manifestations, as delayed diagnosis or misdiagnosis leads to the critical treatment window being missed. Therefore, unexplained abnormal sexual behaviors should raise clinical suspicion for rabies although they are uncommon manifestations of this deadly disease.

## Data Availability

The datasets used during the current study are available from the corresponding author on reasonable request.

## References

[1] Gilbert AT, McCracken GF, Sheeler LL, Muller LI, O'Rourke D, Kelch WJ, New JC (2015). Rabies surveillance among bats in Tennessee, USA, 1996-2010. J Wildl Dis.

[2] Carrara P, Parola P, Brouqui P, Gautret P (2013). Imported human rabies cases worldwide, 1990-2012. PLoS Negl Trop Dis.

[3] WHO expert consultation on rabies (2005). First report (WHO Technical Report Series No. 931; ISBN 92 4 120931 3).

[4] Depani S, Molyneux EM (2012). Case report: an unusual case of priapism. Malawi Med J.

[5] Dutta JK (1996). Excessive libido in a woman with rabies. Postgrad Med J.

[6] Senthilkumaran S, Balamurgan N, Sweni S, Menezes RG (2011). Thirumalaikolundusubramanian P: Hypersexuality in a 28-year-old woman with rabies. Arch Sex Behav.

[7] Bhandari M, Kumar S (1986). Penile hyperexcitability as the presenting symptom of rabies. Br J Urol.

[8] Udwadia ZF, Udwadia FE, Rao PP, Kapadia F (1988). Penile hyperexcitability with recurrent ejaculations as the presenting manifestation of a case of rabies. Postgrad Med J.

[9] Lei YL, Wang XG, Zhang JH, Hu SD (2012). A case report of rabies. Zhejiang J Prev Med (in Chinese).

[10] Tan ZL, Qiang DR (2007). Lessons and Reflection of one case of rabies misdiagnosed. Shanghai J Prev Med (in Chinese).

[11] Jiang S, Chang CD, Wang XG (1986). One case of rabies with rare prodromal symptoms. J Bengbu Med College (in Chinese).

[12] Liu FY (1990). One case of rabies with ejaculation symptoms. Chin J Pract Intern Med (in Chinese).

[13] Zhang ZJ (1995). One case of rabies with special prodromal symptoms. J Clin Derm (in Chinese).

[14] Niu XZ, Qi LH (1993). One case of rabies with frequent spermatorrhea. J Clin Urol (in Chinese).

[15] Xiao LM (1990). One case of rabies with frequent penile erection. J Fujian College Med (in Chinese).

[16] Ge QS, Xiang JL, Xu HR (1992). Two cases of rabies with priapism. J Clin Urol (in Chinese).

[17] Ma GP (1991). One case of rabies with frequent ejaculation and priapism. Zhejiang Med J (in Chinese).

[18] Yang JQ, Wang DY (1998). The first special manifestation of two case of rabies. Clin Med China (in Chinese).

[19] Fu GS, Li ZP, Li YH, Wang JL (1991). The five cases of rabies with special clinical manifestations. Hebei Med J (in Chinese).

[20] Lin SJ (1989). One case of rabies which causes severe spermatorrhea. J Harbin Med Univ (in Chinese).

[21] Feng CG, Jin HC, Yu ZF (1994). One death caused by rabies with paroxysmal severe pain in the pudic. Wuhan Med J (in Chinese).

[22] Du C, Lin Y, Tang Y, Niu HG (1999). One case of rabies with priapism and hypertension. J Luzhou Med College (in Chinese).

[23] Wang FG (1987). One case of rabies which latent for 27 years. J Luzhou Med College (in Chinese).

[24] Tang W (1994). One case of special rabies. J Conting Educ of Physicians (in Chinese).

[25] Yang J, Rile GE, Wang WZ (1990). One case of rabies with special manifestation. Inner Mongolia Med J (in Chinese).

[26] Li JB, Ji QL, Zhang Y (1994). One case of rabies which was misdiagnosed as acute epididymitis. J Linyi Med College (in Chinese).

[27] Xiao JQ, Chen GZ (1992). One case of rabies with early manifestation for frequent priapism and spermatorrhea. J Southeast Univ (in Chinese).

[28] Wu SZ, Zhang W, He MS, Xian ZH (2000). Five cases of rabies with ejaculation as first symptom. J North Sichuan Med College (in Chinese).

[29] Cai MQ, Huang RM (1997). The two cases of rabies with ejaculation as early manifestation. J Med Theory Pract (in Chinese).

[30] Wei ZH (1992). One case of rabies with convulsion in the lower limbs and frequent ejaculation as the first symptom. Youjiang Med J (in Chinese).

[31] Chou Q, Cai M (1991). One case of rabies with low back pain, spermatorrhea and jitter as the first symptom. Sichuang Med J (in Chinese).

[32] Zhan YJ, Yang YD, Jin DX (1991). One case of rabies with spermatorrhea as first symptom. Jiangsu Med J (in Chinese).

[33] Zhong XL (2000). The rabies with penile erection and spermatorrhea as first symptom. Journal of Youjiang Medical University for Nationalities (in Chinese).

[34] He WS (1994). One case of rabies with penile pain as first symptom. Chin J Zoonoses (in Chinese).

[35] Luo XT, Xu YQ (1990). One case of rabies with priapism and frequent spermatorrhea as early manifestation. Jiangxi Med J (in Chinese).

[36] Geng PB (1994). One case of rabies with priapism and frequent ejaculation as first symptom. Anhui Med J (in Chinese).

[37] Zhang SX (1990). One case of rabies with priapism and frequent ejaculation as early manifestation. J New Med (in Chinese).

[38] Wang HC, Tang L, Xie YF (1989). The two cases of rabies with priapism and frequent spermatorrhea as early manifestation. Chin J Pract Intern Med (in Chinese).

[39] Li MQ, Cao XP, Jiang Z (2011). One case of rabies with priapism as first symptom. Chin J Misdiagnostics (in Chinese).

[40] Li GX, Yu XY (2000). One case of rabies with priapism and ejaculation symptom. J New Med (in Chinese).

[41] Daher Ede F, da Silva Junior GB, Ferreira MT, Barros FA, Gurgel TM, Patrocinio RM (2005). Renal involvement in human rabies: clinical manifestations and autopsy findings of nine cases from northeast of Brazil. Rev Inst Med Trop Sao Paulo.

[42] Gardner AM (1970). An unusual case of rabies. Lancet..

[43] Madhusudana SN (1988). Rabies presenting with sexual manifestations. J Indian Med Assoc.

[44] Talaulicar PM (2010). Persistent priapism in rabies. Br J Urol.

[45] Li RY, Sun MS, Zheng ZY (1994). One case of rabies with acute left-sided heart failure as the first symptom. New Med (in Chinese).

[46] Ou Q, Tang XY (2006). Clinical analysis of fifteen dead cases of rabies. Chin J Gen Pract (in Chinese).

[47] Liu JL (2011). Nursing experience of a kid suffered from rabies with priapism as the first symptom. J Chin Modern Nurs.

[48] Qin SB, Du DB, Leng WJ: A case of rabies with priapism and anxiety. A case of rabies with priapism and anxiety 2012; 45:6.

[49] Liu JQ, Tan B, Zhang GL (2013). Diabetic ketosis complicated with rabies-A case report. Chin J Diabetes (in Chinese).

[50] Gao M, Yang B, Yuan JL (2014). A case of rabies with priapism and frequent ejaculation as the first symptoms. Chin J Urol.

[51] Fooks AR, Cliquet F, Finke S, Freuling C, Hemachudha T, Mani RS, Muller T, Nadin-Davis S, Picard-Meyer E, Wilde H (2017). Rabies. Nat Rev Dis Primers.

[52] WHO Expert Consultation on Rabies (2013). Second report. World Health Organ Tech Rep Ser.

[53] Subramaniam Mani R (2016). Human rabies survivors in India: an emerging paradox?. PLoS Negl Trop Dis.

[54] Weyer J, Msimangdermaux V, Paweska JT, Roux KL, Govender P, Coertse J, Markotter W, Nel LH, Blumberg LH (2016). A case of human survival of rabies, South Africa.

[55] de Souza A, Madhusudana SN (2014). Survival from rabies encephalitis. J Neurol Sci.

[56] Karande S, Muranjan M, Mani RS, Anand AM, Amoghimath R, Sankhe S, Belludi AY, Madhusudana SN (2015). Atypical rabies encephalitis in a six-year-old boy: clinical, radiological, and laboratory findings. Int J Infect Dis.

[57] Kumar KV, Ahmad FM, Dutta V (2015). Pituitary cachexia after rabies encephalitis. Neurol India.

[58] Netravathi M, Udani V, Mani RS, Gadad V, Ashwini MA, Bhat M, Mehta S, Chowdhary A, Pal PK, Madhusudana SN (2015). Unique clinical and imaging findings in a first ever documented PCR positive rabies survival patient: A case report. J Clin Virol.

[59] Rawat AK, Rao SK (2011). Survival of a rabies patient. Indian Pediatr.

[60] Mani RS, Anand AM, Madhusudana SN (2016). Human rabies in India: an audit from a rabies diagnostic laboratory. Tropical Med Int Health.

[61] Mani RS, Damodar T, S D, Domala S, Gurung B, Jadhav V, Konanki R, Lingappa L, Loganathan SK, Salagare R (2019). Case reports: survival from rabies: case series from India. Am J Trop Med Hyg..

[62] Willoughby RE, Tieves KS, Hoffman GM, Ghanayem NS, Amlie-Lefond CM, Schwabe MJ, Chusid MJ, Rupprecht CE (2005). Survival after treatment of rabies with induction of coma. N Engl J Med.

[63] Manesh A, Mani RS, Pichamuthu K, Jagannati M, Mathew V, Karthik R, Abraham OC, Chacko G, Varghese GM (2018). Case report: failure of therapeutic coma in rabies encephalitis. Am J Trop Med Hyg.

[64] Huang YC, Harraz AM, Shindel AW, Lue TF (2009). Evaluation and management of priapism: 2009 update. Nat Rev Urol.

[65] Carod-Artal FJ (2018). Infectious diseases causing autonomic dysfunction. Clin Auton Res.

